# Macular thickness changes in a patient with Leber’s hereditary optic neuropathy

**DOI:** 10.1186/s12886-015-0015-1

**Published:** 2015-03-18

**Authors:** Ayako Mizoguchi, Yuki Hashimoto, Yasuhiro Shinmei, Mayo Nozaki, Kan Ishijima, Yoshiaki Tagawa, Susumu Ishida

**Affiliations:** Department of Ophthalmology, Hokkaido University Graduate School of Medicine, N-15, W-7, Kita-ku, Sapporo, 060-8638 Japan; Department of Ocular Circulation and Metabolism, Hokkaido University Graduate School of Medicine, N-15, W-7, Kita-ku, Sapporo, 060-8638 Japan

**Keywords:** Leber’s hereditary optic neuropathy, Spectral domain optical coherence tomography, Ganglion cell complex, Circumpapillary retinal nerve fiber layer

## Abstract

**Background:**

Leber’s hereditary optic neuropathy (LHON) refers to an optic nerve dysfunction due to mutations in the mitochondrial DNA, resulting in visual loss by apoptosis of retinal ganglion cells (RGC). In 20% of LHON cases, their fundus examination looks entirely normal at early stage. There are some reports regarding the circumpapillary retinal nerve fiber layer (cpRNFL) and the ganglion cell analysis around the macula in LHON patients and carriers by using optical coherence tomography.

**Case presentation:**

A 40-year-old female complained of acute visual loss in both eyes. Her best-corrected visual acuity was 0.3 in the right eye and 0.2 in the left eye at the initial visit. Goldmann perimetry revealed bilateral central scotomas. Fundus examination and fluorescein angiography findings were normal, but decreased retinal inner layer thickness was detected around the macular area on spectral domain optical coherence tomography (SD-OCT). One month later, her visual acuity deteriorated to counting fingers in both eyes, and the thinning area of retinal inner layer spread rapidly. Suspected progressive RGC loss led us to check the possibility of LHON, with which the patient was diagnosed due to a positive result for the mitochondrial DNA (mtDNA) 11778 mutation. The ganglion cell complex (GCC) and cpRNFL thicknesses were observed for 24 months by using SD-OCT. The GCC thickness plunged sharply within 3 months followed by gradual decline until 6 months, thereafter showing a plateau up to 24 months. On the cpRNFL map, the temporal quadrant also showed the earliest thinning as seen in the macular area of the GCC map. The thicknesses of the superior, nasal, and inferior quadrants decreased gradually, keeping their normal ranges up to 6 months.

**Conclusions:**

SD-OCT was a useful tool in the diagnosis and follow-up of LHON. The macular GCC thickness map may detect the earliest morphological changes in LHON, as well as the temporal area of cpRNFL, before funduscopic examination reveals optic nerve atrophy.

## Background

Leber’s hereditary optic neuropathy (LHON) is a type of optic atrophy first reported by Theodor Leber in 1871 [1]. Patients usually present with painless subacute or acute, simultaneous or sequential bilateral loss of vision. This maternally inherited genetic disorder with incomplete penetrance is caused by the three most frequent mitochondrial DNA pathogenic mutations (11778/ND4, 3460/ND1, and 14484/ND6) that affect complex I, the first site of the mitochondrial respiratory chain [2]. In LHON, optic atrophy is associated with preferential loss of the central small-caliber optic nerve fibers of the papillomacular bundle, resulting in central scotoma. In the mouse model of LHON, mitochondrial analysis revealed partial complex I and respiration defects and increased reactive oxygen species (ROS) production, whereas synaptosome analysis revealed decreased complex I activity and increased ROS but no diminution of ATP production. Therefore, chronic oxidative stress rather than energy deficiency appears to be a clinically relevant factor in LHON. In the LHON animal model, loss of small and medium retinal ganglion cell (RGC) axons was found in the central and temporal regions of the optic nerve, which corresponds to the human temporal region most affected in LHON [3].

In this study, we monitored retinal thickness by using spectral domain optical coherence tomography (SD-OCT) in an LHON patient for 24 months. Previous OCT studies on LHON evaluated the circumpapillary retinal nerve fiber layer (cpRNFL) [4-8] and the ganglion cell-inner plexiform layer (GCIPL = ganglion cell layer [GCL] + inner plexiform layer [IPL]) around the macula up to 6 months after the onset [9]. Here, we examined the long-term changes of the ganglion cell complex (GCC) thickness (GCC = RNFL + GCL + IPL) from the onset to 24 months as well as cpRNFL thickness from 3 months to 24 months after the onset.

## Case presentation

**Figure 1:**
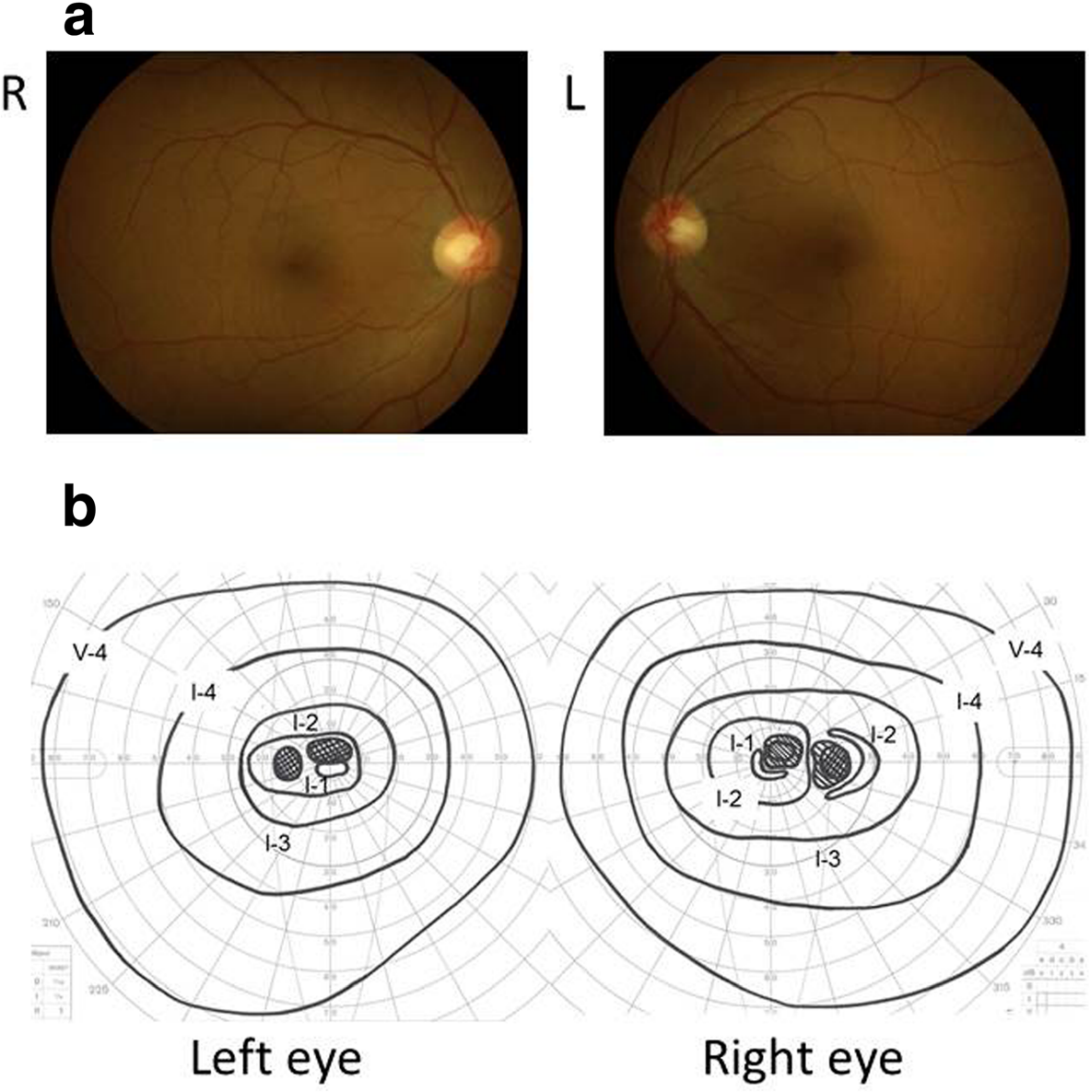
**Fundus photograph (a) and Goldmann perimetry (b).** Fundus appearances looked normal and central scotomas were noted in both eyes at the initial visit.

**Figure 2:**
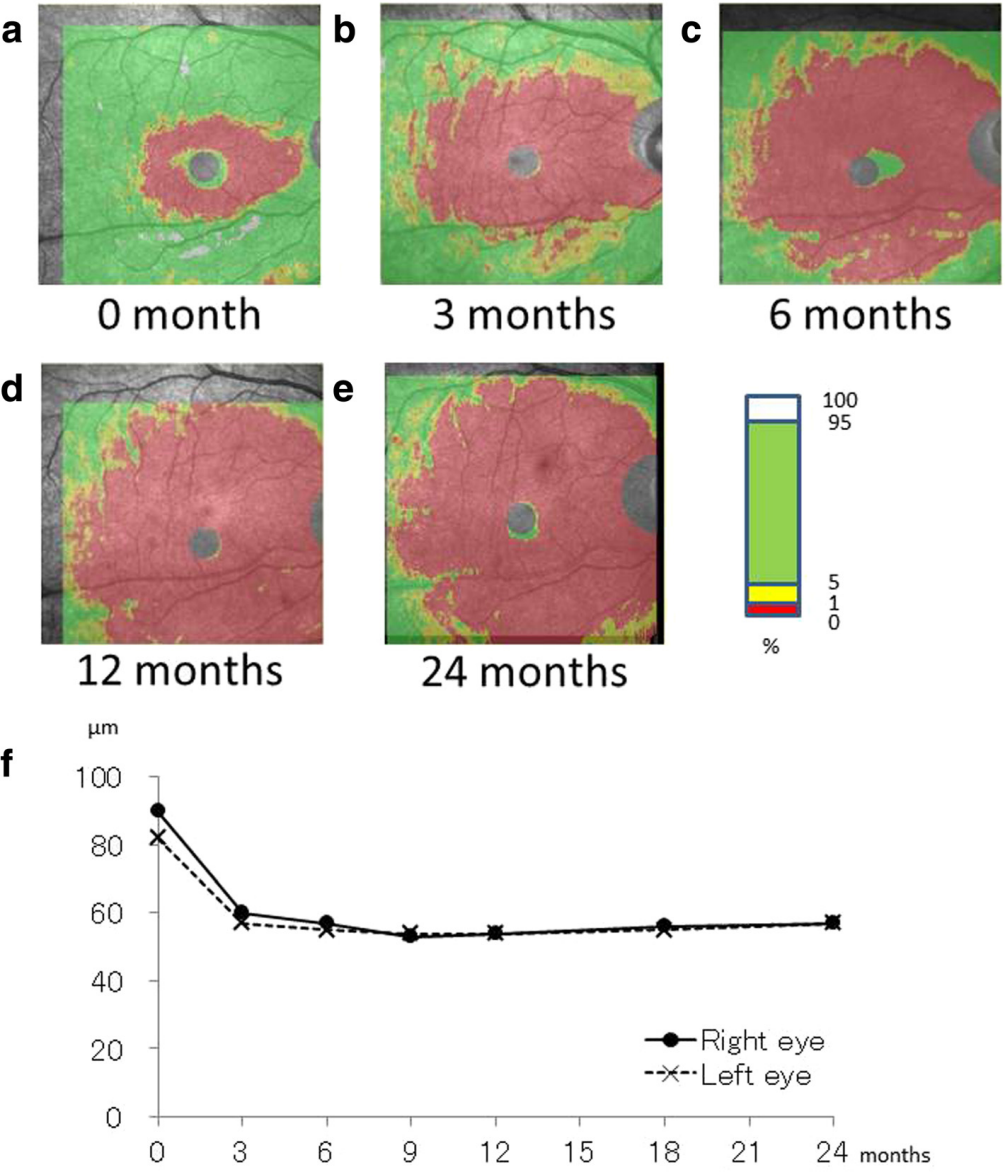
**GCC thickness map.** SD-OCT showing the GCC thickness map in the right eye at initial visit **(a)**, 3 **(b),** 6 **(c)**, 12 **(d)** and 24 **(e)** months after baseline. A graph showing the time course of GCC thickness changes in both eyes **(f)**.

The patient was a 40-year-old female. She presented with acute visual loss in both eyes. She noted that her vision was progressively worsening. She had a history of heavy drinking and smoking, and had alcoholic hepatitis and depressive psychosis. She was in early menopause because she underwent a total hysterectomy combined with bilateral ovary removal for uterine cancer 10 years ago. She had an uncle with low vision. At the first neuro-ophthalmic consultation in our clinic, her best-corrected visual acuity (BCVA) was 0.3 in the right eye and 0.2 in the left eye. Relative afferent pupillary defect was negative and light reflex was appropriate. Slit-lamp biomicroscopy revealed normal findings in the anterior segments. Intraocular pressure was 18 mmHg in the right eye and 15 mmHg in the left eye. Funduscopic examination (Figure 1a) and fluorescein angiography were unremarkable. Goldmann perimetry revealed bilateral central scotomas (Figure 1b). Optic nerve swelling was not detected on magnetic resonance imaging. Visual evoked potentials were non-recordable in both eyes. Thinning of the retinal inner layer was detected on SD-OCT (Figure 2). Blood tests revealed impaired liver function (GOT: 46 IU, GPT: 37 IU, γ-GTP: 423). Folic acid and vitamin B_12_ levels were normal. One month later, her BCVA deteriorated to counting fingers in both eyes, and the thinning area of retinal inner layer spread rapidly. The patient was diagnosed with LHON because of a positive result for the mitochondrial DNA (mtDNA) 11778 mutation. Six months later, the optic disc was observed to be getting paler from the temporal area in both eyes. The visual field continued to deteriorate over a year despite no smoking, reduced alcohol intake, and treatment with vitamin B_12_, vitamin C and ATP disodium salt hydrate.

### OCT findings

We monitored retinal thickness for 24 months by using SD-OCT. We examined the GCC thickness from the onset to 24 months and cpRNFL thickness from 3 months to 24 months after the onset. GCC thickness values were automatically calculated and compared to normative database by the software equipped with SD-OCT (RS-3000 Advance®; Nidek, Gamagori, Japan). The mean GCC thickness was calculated from 8 sectors segmented around the macula (6 mm × 6 mm), which excluded the fovea sector (1 mm × 1 mm). The disc circle scan pattern captures an image of circle with 3.45-mm diameter around the disc and allows cpRNFL thickness analysis compared to the normative database. We studied nasal quadrant (316 – 45-degree unit circle), superior quadrant (46 – 135 degrees), temporal quadrant (136 – 225 degrees), and inferior quadrant (226 – 315 degrees) thicknesses, all of which were automatically calculated by OCT using the built-in software.

Figure 2a-e shows the long-term, sequential changes in GCC thickness in the right eye at initial visit, 3, 6, 12 and 24 months. Thickness map showed that thinning area spread over the whole macular area. The red color zone depicted an extremely thinning area, which represented an abnormal to normal database percentage of < 1%. The green area depicted a relatively normal area evaluated between 5 to 95% in a population of normal eyes. The red zone on the color-coded normative database map of the GCC continued to expand until 12 months when the most of the macular area turned red. Figure 2f shows the time course of GCC thickness changes in both eyes. The GCC thickness plunged sharply within 3 months followed by gradual decline until 6 months, thereafter showing a plateau up to 24 months.

**Figure 3:**
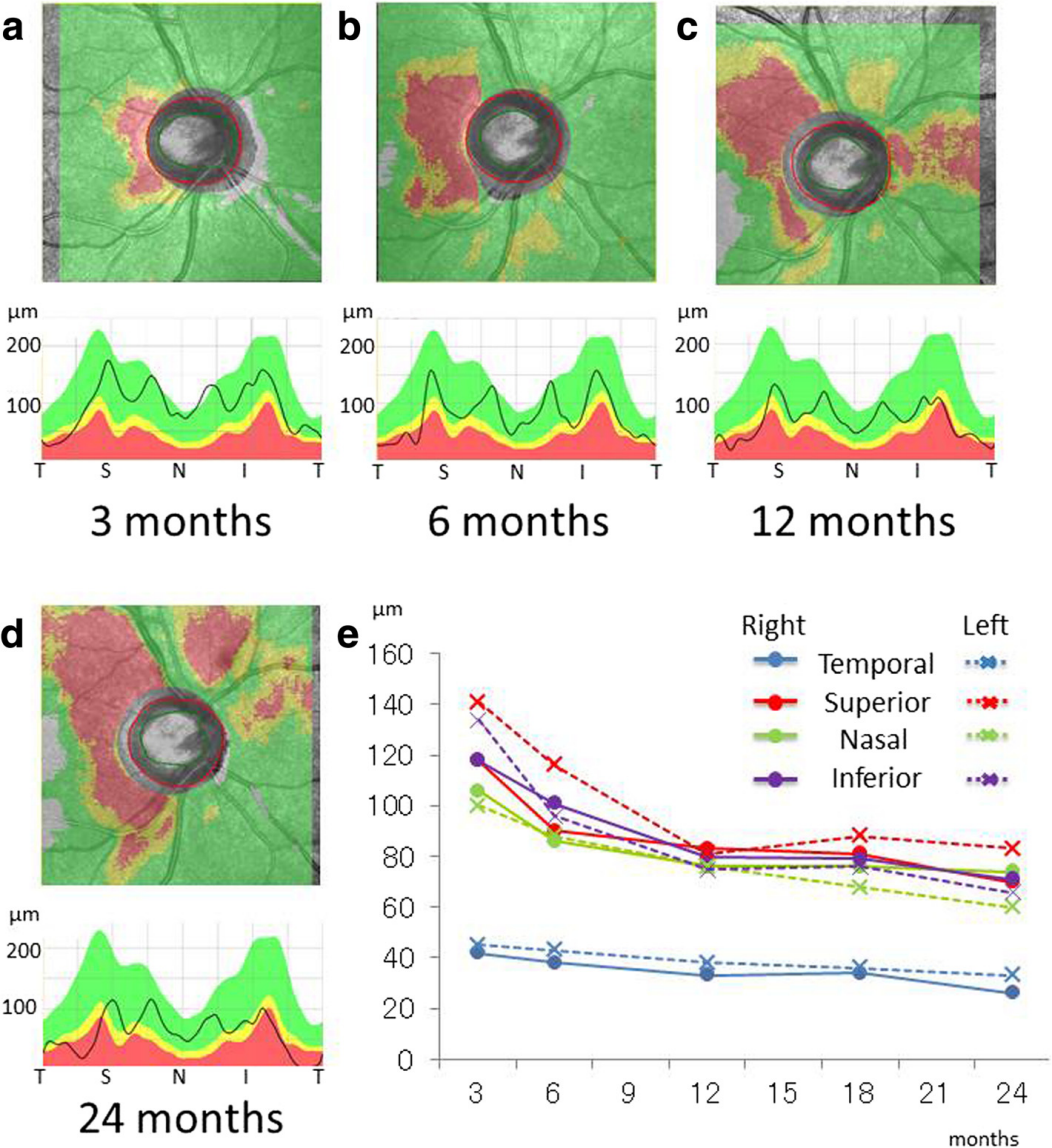
**cpRNFL thickness map.** SD-OCT showing the cpRNFL thickness map (top) and TSNIIT graph (bottom) in the right eye at 3 **(a)**, 6 **(b)**, 12 **(c)** and 24 **(d)** months after the initial visit. A graph showing the time course of cpRNFL thickness changes in both eyes **(e)**.

Figure 3a-d shows the combined images of RNFL thickness maps (top) and temporal-superior-nasal-inferior-temporal (TSNIT) graphs (bottom) at 3, 6, 12 and 24 months. Figure 3e shows the long-term, sequential changes in RNFL thickness of both eyes for each quadrant (temporal, superior, nasal and inferior). The changes in RNFL thickness were equivalent for both eyes in all quadrants throughout the two-year follow-up period. The temporal quadrant exhibited the earliest thinning at 3 months as seen in the macular GCC thickness changes. In the superior, nasal, and inferior quadrants, thickness changes were more evident on the later stage maps even after 12 months. The cpRNFL thickness also revealed that the latent thickening of RNFL continued earlier in these quadrants except the temporal one before we noticed disc atrophy by funduscopic examination at 6 months (Figure 3e).

## Discussion

Classical LHON cases are characterized by several distinct abnormalities including vascular tortuosity of the central retinal vessels, swelling of the RNFL, and a circumpapillary telangiectatic microangiopathy [10]. In about 20% of LHON cases, however, the optic disc looks entirely normal, leading to a misdiagnosis such as functional vision loss [11-13]. Previous studies using OCT in LHON patients showed that increased RNFL thickness around the optic disc first appeared in the temporal and inferior quadrants but was more evident in the superior and nasal quadrants at 3 months after the onset. These findings were considered consistent with the established preferential early involvement of the papillomacular bundle in LHON [5]. In the present LHON patient, indeed, the only temporal quadrant of cpRNFL became thinner than normal controls until 6 months. In contrast, the other quadrants (superior, inferior, and nasal) became thinner than normal controls after 6 months. The mean RNFL thickness decreased to the baseline value at 6 months because of early optic nerve atrophy, and RNFL thinning progressed until optic nerve atrophy was established. Although our first examination might be somewhat later than others, we obtained the equivalent result that the temporal retina thinned first of all.

Akiyama et al. suggessted that ganglion cell analysis mesuring the thickness of the macular GCIPL (GCL + IPL) could precisely detect the time-dependent loss of retinal ganglion cells in LHON eyes during 6-month follow-up while RNFL thickness had not decreased yet [9]. In our case, the GCC thickness map detected the earliest thinning of the GCC around the fovea at the initial visit (Figure 2a) despite no remarkable appearances on funduscopic and angiographic examinations. When the small central scotomas were detected on Goldmann perimetry, the GCC thinning had already developed around the fovea. These findings suggest that GCC thickness measurements are effective to discriminate between funduscopically normal LHON and functional vision loss.

Previous histopathological studies on LHON eyes also showed a dramatic loss of RGCs [14,15]. The mtDNA content at the macular (temporal) region is higher than that at the nasal region. The macular-to-nasal ratio was shown to be even higher in the carrier of LHON than in healthy controls [16]. The GCC thickness map, which more directly reflects macular RGC loss, is a viable alternative to cpRNFL analysis for the diagnosis and follow-up of LHON patients. In our case, indeed, the progressive thinning of the GCC was observed to be acute and severe. This may be related to the lack of visual recovery in the present patient with the mtDNA 11778 mutation, which is known to cause the poorest outcome for vision among the LHON mutations.

Much attention should be paid, however, to the atypical and peculiar features of the present 11778 LHON case. These include female onset, lack of optic disc microangiopathy and fiber swelling, and a past history of heavy drinking and smoking. We could not deny the possibility that each of these aspects could affect and modify the pattern of RGC loss seen in our patient. Future studies on additional cases are needed to further establish the significance of the GCC evaluation for the management of LHON.

## Conclusions

We observed long-term, sequential changes in the thickness of both GCC and cpRNFL in a LHON patient by using SD-OCT for two years. The GCC and the temporal RNFL thickness changes sharply reflected the acute loss of RGCs. In contrast, the other quadrants of cpRNFL (superior, inferior, and nasal) demonstrated gradual change with funduscopically detectable optic nerve atrophy at 6 months and thereafter. Accordingly, much attention should be paid to the dissociation between the GCC and cpRNFL changes especially at the early stage of LHON.

### Consent

Written informed consent was obtained from the patient for publication of this case report and any accompanying images. A copy of the written consent is available for review by the Editor-in-Chief of this journal.

## Abbreviations

LHON: Leber’s hereditary optic neuropathy
RGC: Retinal ganglion cell
cpRNFL: Circumpapillary retinal nerve fiber layer
GCC: Ganglion cell complex
OCT: Optical coherence tomography
SD-OCT: Spectral domain OCT
mtDNA: mitochondrial DNA
ROS: Reactive oxygen specie
ATP: Adenosine triphosphate
GCIPL: Ganglion cell-inner plexiform layer
GCL: Ganglion cell layer
IPL: Inner plexiform layer
RNFL: Retinal nerve fiber layer
BCVA: Best-corrected visual acuity
TSNIT: Temporal-superior-nasal-inferior-temporal
